# Case Report: Three-Dimensional Printing Model for Surgical Planning of Left Ventricular Aneurysm: Evolution Toward Tailoring Surgery

**DOI:** 10.3389/fcvm.2022.852682

**Published:** 2022-03-25

**Authors:** Nazario Carrabba, Francesco Buonamici, Rocco Furferi, Monica Carfagni, Matteo Vannini, Renato Valenti, Alfredo Giuseppe Cerillo, Niccolò Marchionni, Pierluigi Stefàno

**Affiliations:** ^1^Cardiovascular and Thoracic Department of Careggi Hospital, Florence, Italy; ^2^Department of Industrial Engineering of Florence, University of Florence, Florence, Italy

**Keywords:** 3D printing model, left ventricular aneurysm, CAD, heart failure, surgical ventricular restoration

## Abstract

A 59-year-old woman was admitted to the emergency department for heart failure (HF), New York Heart Association (NYHA) IV, showing an anterior, evolved myocardial infarction (MI) with a wide apical left ventricular aneurysm (LVA), ejection fraction (EF) 24%, and global longitudinal strain (GLS) −5. 5% by echo. Cardiac magnetic resonance imaging (MRI) confirmed an apical LVA without thrombus, EF 20%, and a transmural delayed enhancement in the myocardium wall. Coronarography showed a three-vessel disease with occluded proximal left anterior descending (LAD) and proximal right coronary artery (RCA). Based on the cardiac CT scan, we decided to generate a three-dimensional (3D) print model of the heart, for better prediction of residual LV volumes. After LVA surgery plus complete functional revascularization, an optimal agreement was found between predicted and surgical residual LV end-diastolic (24.7 vs. 31.8 ml/m^2^) and end-systolic (54.1 vs. 69.4 ml/m^2^) volumes, with an improvement of NYHA class, from IV to I. The patient was discharged uneventfully and at 6- and 12-month follow-up, the NYHA class, and LV volumes were found unchanged. This is a second report describing the use of the 3D print model for the preoperative planning of surgical management of LVA; the first report was described by Jacobs et al. among three patients, one with a malignant tumor and the remaining two patients with LVA. This article focused on the use of the 3D print model to optimize surgical planning and individualize treatment of LVA associated with complete functional revascularization, leading to complete recovery of LV function with a favorable outcome.

## Introduction

After myocardial infarction (MI), patients with severe heart failure (HF) carry a poor prognosis, and despite major advances, long-term medical management alone might be insufficient (1). Cardiac transplantation and ventricular assist devices are definitive or temporary surgical therapies. Although it has been shown that mitral valve (MV) and surgical coronary revascularization (CR) can lead to the improvement of symptoms in left ventricular aneurysm (LVA) patients, ventricular dilation and dysfunction might be so advanced that surgical reconstruction of the LV geometry must be attempted. We herein report a patient with LVA with severe systolic dysfunction, who underwent surgical LV reshaping using a 3D print model with the subsequent optimal recovery of systolic function (2).

## Case Presentation

**Figure 1:**
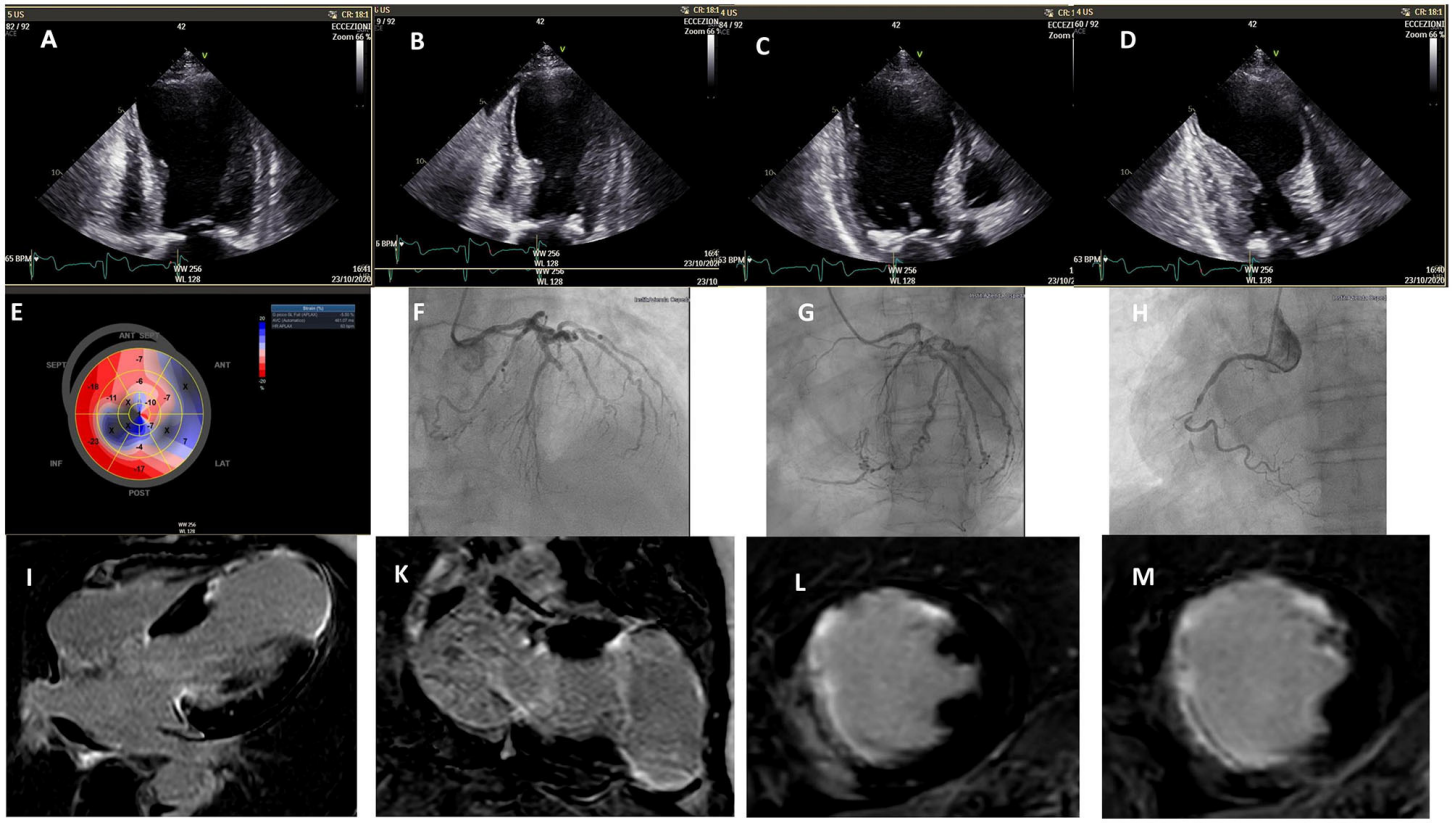
2D echo: **(A–D)** left ventricular aneurysm: systolic and diastolic phase; **(E)** baseline global longitudinal strain; coronary angiograms; **(F)** occlusion of the proximal left anterior descending coronary artery; **(G)** circumflex artery; **(H)** occlusion of proximal right coronary artery; cardiac MRI; **(I–M)** transmural delayed enhancement in the myocardium wall supplied by LAD.

Our patient was a 59-year-old woman admitted to the emergency department for HF, New York Heart Association (NYHA) IV, with dyspnea that started 10 days before. She was a former smoker, affected by hypercholesterolemia and hypertension, on angiotensin-converting enzyme (ACE) inhibitor treatment at the time of presentation. We started management of HF using high doses of loop diuretics i.v., beta blockers, ACE inhibitors, mineralocorticoids, statins, and aspirin. Routine blood investigations were notable for NT-proBNP 6,526 ng/dl and troponin-hs 196 ng/l. An anterior, evolved MI infarction was revealed by ECG, and a two-dimensional (2D)-echocardiogram showed a wide apical LVA (6 × 5.5 cm) without thrombus, EF 24% and global longitudinal strain (GLS) 5.5% (Figures 1A–E and Supplementary Video 1), and mild mitral regurgitation. The coronary angiogram showed a 60% stenosis of proximal left main and occlusion of proximal left anterior descending (LAD) as well as occlusion of proximal right coronary artery (RCA) (Figures 1F–H). To better evaluate LVA, further imaging was advised. Cardiac MRI confirmed an apical LVA without thrombus, EF 20%, and a transmural delayed enhancement in the myocardium wall, supplied by LAD, specifically, in the middle and apical segment of the septum, the distal segment of the anterior wall, and apical segments of LV, with microvascular obstruction observed in the septum (Figures 1I–M). Since the 3D volumetric sequences were not planned in an MRI protocol, to optimize surgical planning, based on the cardiac CT scan, we decided to generate a 3D print model of the LVA for a better prediction of the residual surgical LV volumes, LVA volume (47%), and the geometrical shape of MV, annulus, and papillary muscle attachment (Table 1). A dedicated contrast-enhanced CT scan (high pitch, dual source) was made according to the following protocol: retrospective ECG-gated spiral acquisition, with tube modulation. Thin reconstructions (0.4 mm) were exported into imaging processing software version 22.0 (Materialize Mimics Medical, Leuven, Belgium) to delineate and segment the left-sided structures, including the MV, annulus, and leaflets. All the phases were available, and diastolic and systolic phases were chosen for LV reconstruction. Patient-specific 3D models were then printed using tissue rigid material for left chambers. The models were printed using Fused Filament Fabrication technology; specifically, a Makerbot Replicator + machine was used to produce plastic models in the PLA material. A 0.1 mm layer height was selected during fabrication, to ensure valid resolution for the application. The cardiologist and cardiac surgeon used the 3D-printed models during their encounters with the patient, to illustrate the specific pattern of LVA and anticipated repair results. The patient expressed a markedly improved understanding of her LVA and planned operation.

**Table 1:** Left ventricular volumes before, planned, and after surgery.

	**LV-EDV**	**LV-ESV**	**SV**	**EF (%)**
**3D-printed model**				
Before surgery (mL/m^2^)	101.8	77.1	24.7	24
Planned (mL/m^2^)	54.1	24.7	29.4	54
After surgery (mL/m^2)^	69.4	31.8	37.3	54

*LV, left ventricular; EDV, end diastolic volume; ESV, end systolic volume; SV, stroke volume; EF, ejection fraction*.

**Figure 2:**
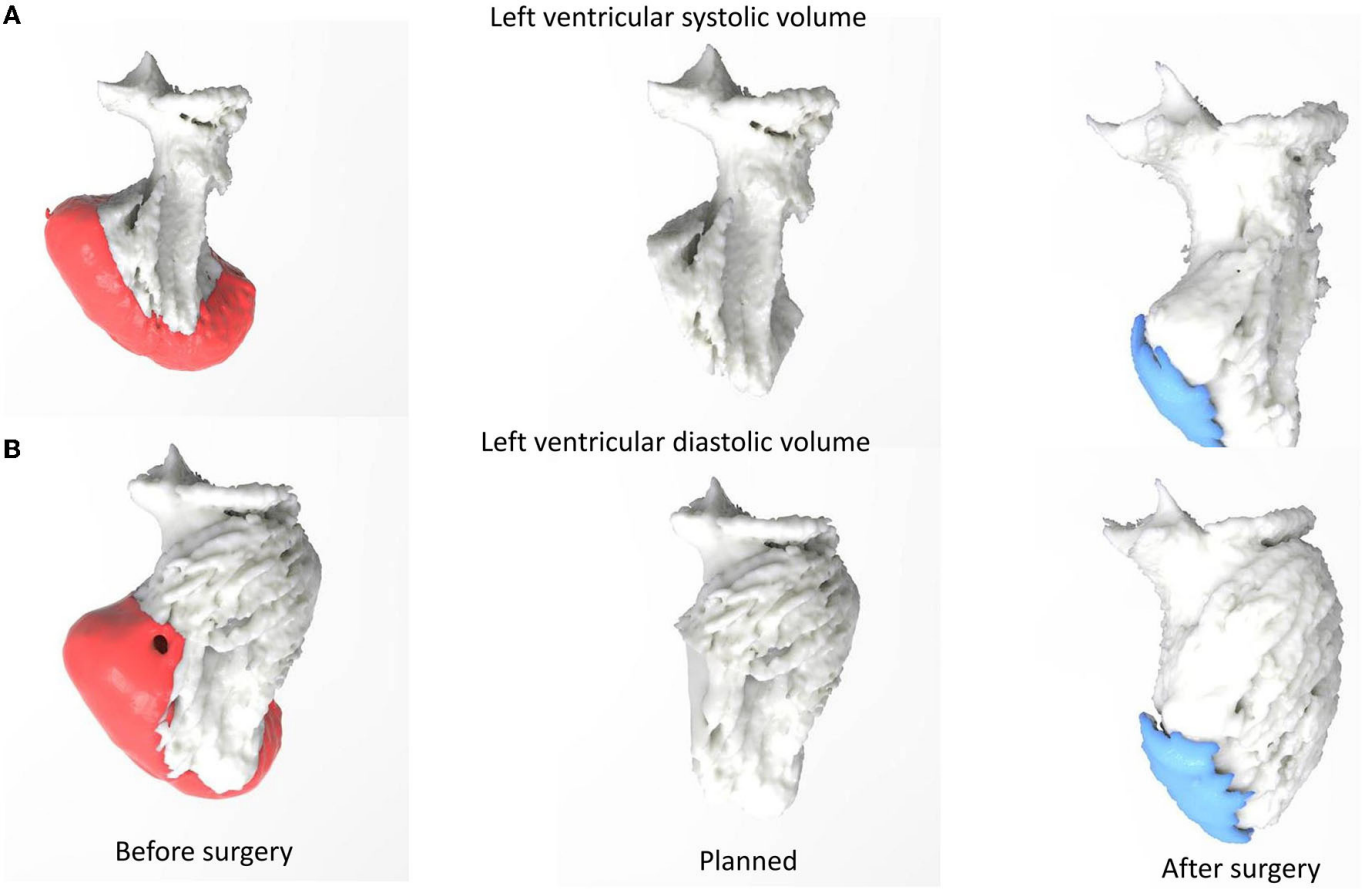
3D print model: **(A)** left ventricular systolic volume, **(B)** left ventricular diastolic volume before, planned, and after surgery.

**Figure 3:**
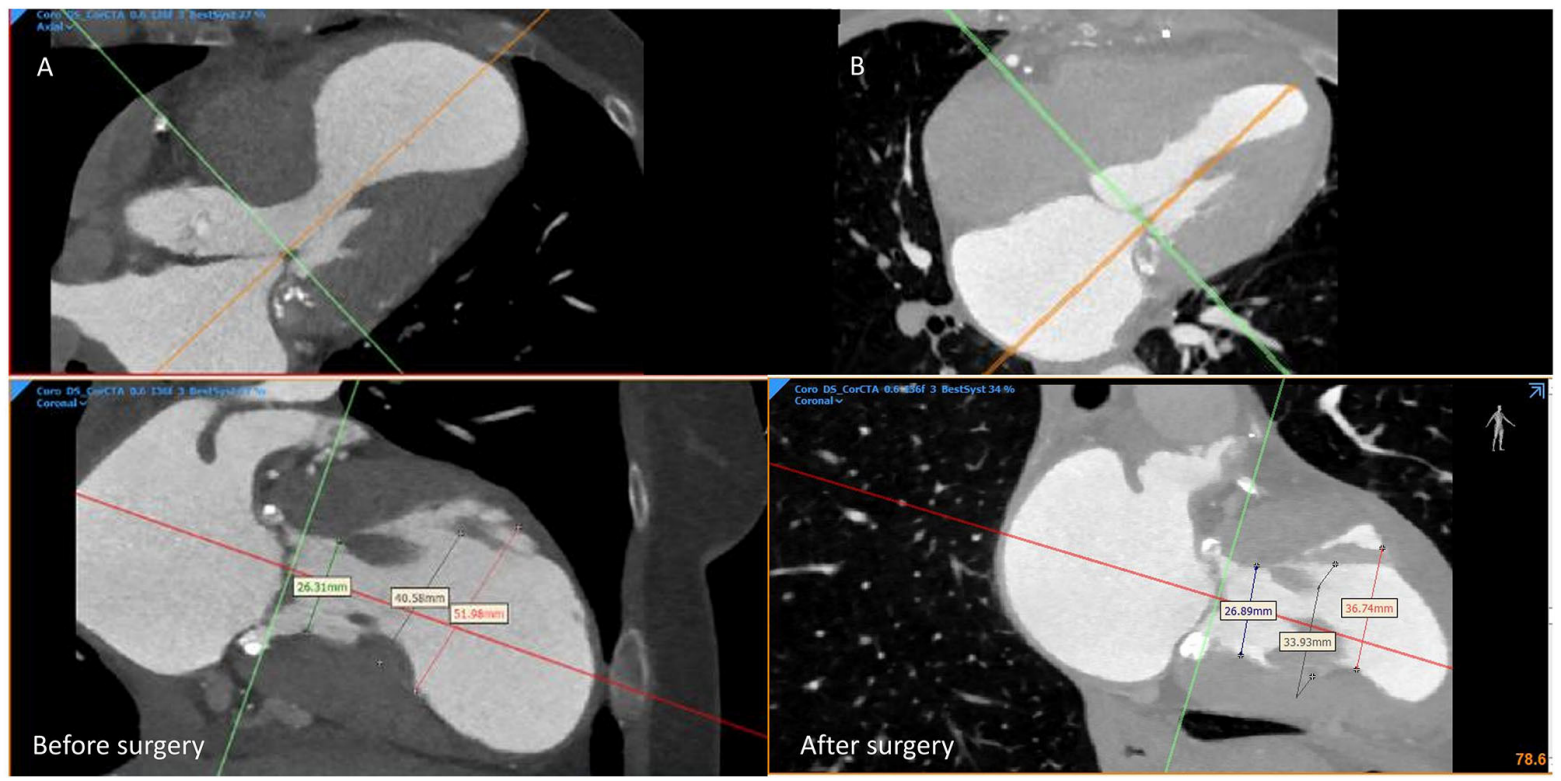
CT scan: the geometrical shape of the mitral valve, annulus, and papillary muscle attachment **(A)** before surgery, **(B)** after surgery.

After heart team discussion, the surgical ventricular restoration (SVR) of the LV chamber appeared as an attractive strategy, combined with complete CR. The risk in generating a small residual LV was discussed, as it is not trivial. To optimize surgical planning, we used a 3D print model of the LVA for a better prediction of the residual surgical LV volumes. The patient underwent SVR excluding dyskinetic portions of the anterior wall and septum, reshaping the LV with a stitch that encircled the transitional zone between contractile myocardium and aneurysmal tissue, and using a “Gore-Tex” patch to reestablish ventricular wall continuity. A complete functional CR was also attempted, using the left anterior internal mammary artery for the marginal branch and saphenous graft for RCA. The operation improved the size and geometry of the left ventricle, reduced wall tension and paradox movement, and enhanced overall systolic function, EF 54% (Figures 2A,B), and also GLS (average −14) and LV torsion (3.6, 1.3–2.1 deg/cm) (Supplementary Videos 2–4). In addition, the geometry of papillary muscle was preserved, without significant change in the interpapillary muscle distance and tenting height (Figures 3A,B), with a trivial residual mitral regurgitation (MR). Importantly, an optimal agreement was found between the predicted and surgical residual LV volumes (Table 1), with an improvement of NYHA class from IV to I. The patient remained uneventful and was discharged 7 days after surgery. At 6- and 12-month follow-up, the patient remained in NYHA class I, and LV volumes were found unchanged.

## Discussion

Heart failure is a major health problem, with increasing prevalence due to the aging of the population and the increased survival after MI. Patients with severe HF have a poor prognosis, and despite major advances, long-term medical management alone may be insufficient. Cardiac transplantation and ventricular assist devices are permanent or temporary surgical therapies (3, 4). After an MI, the LVA is located at the apex or in the anterior wall in 90% of cases, and in the posterior–inferior wall in the remaining 10%. Although it has been shown that MV and surgical CR can lead to the improvement of symptoms in LVA patients, ventricular dilation and dysfunction might be so advanced that surgical reconstruction of the LV geometry must be attempted (5). Certainly, the concept of reducing LV volume to improve global contractile function is not new. Currently, the remodeling of the LVA is performed mostly with the surgical techniques developed by Dor and Jatene (6). The STICH trial did not show improved survival with surgical CR associated with SVR in comparison to CR alone (7). However, a *post hoc* analysis of that trial showed that a postoperative LVESVI of 70 ml/m^2^ or lower resulted in improved survival, compared with CR alone (8). In agreement with these results, the most recent ESC/EACTS guidelines recommend SVR in combination with CR in selected HF patients with a scar in the LAD territory, especially, if a postoperative LVESVI below 70 ml/m^2^ can be predictably achieved (class of recommendation IIb; level of evidence B) (9). In addition, when the removal of LVA, in comparison with the baseline LVESVi, is <30%, the beneficial effect of this surgical intervention could be lower than that expected, and in this case, the surgical intervention would be contraindicated (8). Beyond the surgeon's experience, optimal pre-procedural planning based on imaging is of pivotal importance to achieve a more detailed analysis of the heart structures and accurate measurements of LV volumes (10). Volumetric image acquisition plays a critical role in 3D printing. Not only does it determine the geometric accuracy of the 3D model, but also it characterizes tissue properties and directs the choice of the appropriate printing materials. A number of modern cardiovascular imaging techniques have been used to acquire 3D image data for 3D printing. Contrast-enhanced multidetector row CT with electrocardiographic gating/triggering has been the most commonly used imaging modality for 3D printing because of its fast acquisition, superb spatial resolution, and excellent ability of tissue characterization differentiating metal implants and calcific lesions from soft tissues. The temporal resolution of modern CT varies in the range of 75–200 ms, depending on its make and model. Compared with CT, 3D cardiac magnetic resonance has a relatively lower spatial resolution and longer acquisition time. However, because of the absence of ionizing radiation, the 3D cardiac MRI with free-breathing technique has been frequently used in modeling the structures of the cardiac chambers and great vessels in pediatric patients and young adults for 3D printing. On the other hand, because of the wide availability, high temporal resolution, and ease of performing echocardiography at the bedside, echocardiography has been used to acquire images for 3D printing, especially for imaging of valve disease. The main limitation of 3D echocardiography is the relatively low signal-to-noise ratio, which makes image post-processing and 3D modeling more challenging. Furthermore, because of the tradeoff between the size of the acoustic window and the spatiotemporal resolution, 3D modeling of the complete heart anatomy using echocardiography remains difficult. The usefulness of 3D models is particularly felt in cardiac surgery and interventional cardiology, where a detailed knowledge of patient-specific anatomy is fundamental to guide procedures (10). Indeed, the possibility of obtaining a 3D virtual model of the heart structure and physically printing the hollow heart anatomy introduces a radical revolution in interventional planning and in surgeons' training. In the treatment of LVA, for instance, the use of the 3D print model may help the surgeon accurately predicting LVESVI avoid the risk of a restrictive pattern or a distorted ventricular shape generating a severe MR. The 3D-printed model has been applied in the field of medical education and training, procedural planning and simulation, device innovation, and also communication to the patients (11). From a surgeon's and interventional cardiologist's perspective, printed models add 3D spatial and tactile dimensions to a patient's specific cardiovascular disease, thus, leading to improved 3D conceptualization and enhanced visuospatial skills (12). The ultimate goals are to optimize pre-procedural planning, to tailor the procedure to the patient's specific anatomy, to be prepared for possible complications, and to shorten operative and extracorporeal circulation times, reducing postoperative multiorgan dysfunction. From the patient's perspective, personalized 3D models have the potential to alleviate anxiety about the upcoming procedure by improving understanding of the disease and the anticipated operation, and by enhancing the patient–doctor relationship and communication.

## Conclusion

Nowadays, a personalized surgical approach is mandatory to satisfy the different needs of post-infarct LV remodeling. Patient-specific 3D-printed models can be used for a comprehensive assessment of the LVA, pre-procedural planning, and patient education, translating into excellent results. Optimal planning of SVR is of crucial relevance in preventing a restrictive pattern or a distorted ventricular shape, potentially translating into poor outcomes. Based on a 3D-printing model, it is conceivable that, in the near future, a tailored approach to different phenotypes of LVA will make the planning of the most appropriate residual LV volumes possible in the individual patient.

## Data Availability Statement

The raw data supporting the conclusions of this article will be made available by the authors, without undue reservation.

## Ethics Statement

The studies involving human participants were reviewed and approved by Careggi Hospital EC. The patients/participants provided their written informed consent to participate in this study.

## Author Contributions

NC: substantial contributions to the conception or design of the work, agree to be accountable for all aspects of the work in ensuring that questions related to the accuracy or integrity of any part of the work are appropriately investigated and resolved, and drafting the work or revising it critically for important intellectual content. FB: substantial contributions to the conception or design of the work and agree to be accountable for all aspects of the work in ensuring that questions related to the accuracy or integrity of any part of the work are appropriately investigated and resolved. RF and MC: substantial contributions to the conception or design of the work and provide approval for publication of the content. MV, RV, and AC: substantial contributions to the conception or design of the work, or the acquisition, analysis, or interpretation of data for the work. NM and PS: substantial contributions to the conception or design of the work and provide approval for publication of the content. All authors contributed to the article and approved the submitted version.

## Conflict of Interest

The authors declare that the research was conducted in the absence of any commercial or financial relationships that could be construed as a potential conflict of interest.

## Publisher's Note

All claims expressed in this article are solely those of the authors and do not necessarily represent those of their affiliated organizations, or those of the publisher, the editors and the reviewers. Any product that may be evaluated in this article, or claim that may be made by its manufacturer, is not guaranteed or endorsed by the publisher.

## Supplementary Material

Supplementary Video 1 2D (2- 4-chamber and short axis view) and 3D echo of left ventricle aneurysm before surgery.

Supplementary Video 2 Time-volume curve of left ventricular and ejection fraction after surgery.

Supplementary Video 3 Left ventricular global longitudinal strain after surgery.

Supplementary Video 4 Left ventricular torsion after surgery.

## References

[1] Bahit MC Kochar A Granger CB. Post-myocardial infarction heart failure. JACC Heart Fail. (2018) 3:179–86. 10.1016/j.jchf.2017.09.015 29496021

[2] Jacobs S Grunert R Mohr FW Falk V. 3D imaging of cardiac structures using 3D heart models for planning in heart surgery: a preliminary study. Interact. Cardiovasc Thorac Surg. (2008) 7:6–9. 10.1510/icvts.2007.156588 17925319

[3] Rose EA Gelijns AC Moskowitz AJ Heitjan DF Stevenson LW. The randomized evaluation of mechanical assistance for the treatment of congestive heart failure (REMATCH) study group. Long-term use of a left ventricular assist device for end- heart failure. N Engl J Med. (2001) 20:1435–43. 10.1056/NEJMoa012175 11794191

[4] Moss AJ Zareba W Hall WJ Klein H Wilber DJ Cannom DS. The multicenter automatic defibrillator implantation trial ii investigators. Prophylactic implantation of a defibrillator stage in patients with myocardial infarction and reduced ejection fraction. N Engl J Med. (2002) 12:877–83. 10.1056/NEJMoa013474 11907286

[5] Alderman EL Fisher LD Litwin P Kaiser GC Myers WO Maynard C. Results of coronary artery surgery in patients with poor left ventricular function (CASS). Circulation. (1983) 68:785–95. 10.1161/01.CIR.68.4.785 6352078

[6] Dor V Saab M Coste P Kornaszewska M Montiglio F. Left ventricular aneurysm: a new surgical approach. Thorac Cardiovasc Surg. (1989) 37:11–9. 10.1055/s-2007-1013899 2522252

[7] Jones RH Velazquez EJ Michler RE. STICH Hypothesis 2 investigators. Coronary bypass surgery with or without surgical ventricular reconstruction. N Engl J Med. (2009) 360:1705–17. 10.1056/NEJMoa0900559 19329820 PMC3265934

[8] Michler RE Rouleau JL Al-Khalidi HR. STICH Trial Investigators. Insights from the STICH trial: change in left ventricular size after coronary artery bypass grafting with and without surgical ventricular reconstruction. J Thorac Cardiovasc Surg. (2013) 146:1139–45. 10.1016/j.jtcvs.2012.09.007 23111018 PMC3810307

[9] Windecker S Kolh P Alfonso F Collet JP Cremer J Falk V. 2014 ESC/EACTS Guidelines on myocardial revascularization: the Task Force on Myocardial Revascularization of the European Society of Cardiology (ESC) and the European Association for CardioThoracic Surgery (EACTS) Developed with the special contribution of the European Association of Percutaneous Cardiovascular Interventions (EAPCI). Eur Heart J. (2014) 35:2541–619. 10.1093/eurheartj/ehu278 25173339

[10] Harb SC Rodriguez LL Vukicevic M Kapadia SR Little SH. Three-dimensional printing applications in percutaneous structural heart interventions. Circ Cardiovasc Imaging. (2019) 12:e009014. 10.1161/CIRCIMAGING.119.009014 31594408

[11] Bartel T Rivard A Jimenez A Mestres CA Müller S. Medical three-dimensional printing opens up new opportunities in cardiology and cardiac surgery. Eur Heart J. (2018) 39:1246–54. 10.1093/eurheartj/ehx016 28329105

[12] Wang DD Qian Z Vukicevic M. 3D Printing, Computational Modeling, and Artificial Intelligence for Structural Heart Disease. J Am Coll Cardiol Img. (2021). 14:41–60. 10.1016/j.jcmg.2019.12.022 32861647

